# Effect of Rapid Weight Loss on Hydration Status and Performance in Elite Judo Athletes

**DOI:** 10.3390/biology11040500

**Published:** 2022-03-24

**Authors:** Bayram Ceylan, Latif Aydos, Jožef Šimenko

**Affiliations:** 1 Coaching Education, Faculty of Sport Sciences, Kastamonu University, 37150 Kastamonu, Turkey; 2 Physical Education and Sport, Faculty of Sport Sciences, Gazi University, 06560 Ankara, Turkey; latifaydos@gmail.com; 3 Essex Pathways Department, University of Essex, Colchester CO4 3SQ, UK

**Keywords:** combat sports, dehydration, judo performance, weight regain, rules

## Abstract

**Simple Summary:**

This study investigated the effect of 5% rapid weight loss on hydration status and performance in elite judo athletes. The study demonstrated that rapid weight loss performed in 48 h induced dehydration and increased heart rate responses during the exercise. However, it did not negatively affect performance during judo-specific tasks. Despite this finding, rapid weight loss may lead to hazardous effects on the health and performance of judo athletes in the long term. Thus, more precautions should be taken to stop judo athletes from resorting to rapid weight loss before competitions with better in-season weight management.

**Abstract:**

Background: This study aimed to investigate the effect of 5% rapid weight loss on hydration status and judo performance in highly trained judo athletes. Methods: Eighteen male judo athletes participated in the study and were divided into two groups: control and rapid weight loss (RWL). RWL athletes were given 48 h to cut 5% of their body mass while the control group followed their routines. Athletes performed three measurements, including hydration, body mass and three consecutive special judo fitness tests (SJFTs). At the 1st and 6th minutes following each SJFT and 1st, 6th and 15th minutes following the last SJFT, blood lactate and heart rate (HR) was monitored. Results: The effect of RWL on variables was tested with split-plot ANOVA. RWL significantly affected urine specific gravity with a higher value following weight loss compared to baseline and recovery (F2-32 = 13.2, *p* < 0.001). In addition, athletes’ SJFT total throw numbers differed among measurements (F2-32 = 7.70, *p* < 0.001). Athletes presented worse SJFT index after weight loss (F2-32 = 8.05, *p* = 0.01; F1-16 = 6.43, *p* = 0.02, respectively). HR changed significantly among measurements days and times (F28-448 = 143.10, *p* < 0.001). Conclusion: RWL induced dehydration and impaired heart rate recovery in highly trained judo athletes, and they could not rehydrate between competition simulated weigh-in and 15 h of recovery.

## 1. Introduction

Judo is a grappling Olympic combat sport where athletes compete according to weight categories [1]. The weight categories aim to maximize competition fairness and decrease injury risks [2]. Nevertheless, judo athletes, like many other combat sports athletes, resort to rapid weight loss (RWL) and rapid weight gain (RWG) to have an advantage against lighter and weaker opponents [3,4]. While some studies show that it helps athletes gain an advantage during competition [4,5], other studies revealed no improvement in competitive performance [6,7]. In many combat sports such as judo, mixed martial arts (MMA), boxing, wrestling and taekwondo, most of the athletes apply rapid weight loss [8,9]. Specifically, in judo, 60–80% of the athletes resort to rapid weight loss [8,10], and they start weight cutting at very early ages [10,11], which has been stated to negatively affect development during puberty and increase health-related risks [10,12].

There are conflicting findings in the literature related to the effects of RWL in combat sports. Some studies suggested that RWL resulted in impaired repeated aerobic and anaerobic performance and a decrease in strength gain [12,13,14], while other studies presented no negative effects of RWL on aerobic and anaerobic performance and repeated efforts [15,16,17]. There are also conflicting results related to RWG in combat sports. Some studies stated RWG is an effective strategy, while others stated it does not improve performance during competition [6,7]. Reale et al. [5] investigated the effect of RWG on success during an international judo competition and indicated that medal winners regained more weight following the weigh-in than non-medalists, thus suggesting RWG provides an advantage. However, boxers with higher body mass gain following official weigh-in were reported not to present better results [7]. However, direct comparison between boxing and judo should not be done, due to different external loads between punching and kicking sports (karate, boxing, taekwondo, etc.) in comparison to grappling and throwing sports (judo, wrestling, etc.).

RWL induces dehydration in combat sports athletes. Dehydration may cause physiological changes in the body, diminishing exercise performance and putting one’s health at risk [18]. Previous research suggest that dehydration cause impaired aerobic and anaerobic performance, impaired skill-based performance and impaired thermoregulation and cardiovascular stress [18,19]. Given that judo competitions are spread throughout the year under the IJF World Tour [1], athletes are exposed to RWL and thus extreme dehydration [20]. Moreover, previous research highlighted that judo athletes remain in a dehydrated state in competitive and training environments in different age categories despite having the opportunity to consume fluids or rehydrate before official competitions [20,21,22,23]. Moreover, judo athletes adopt dehydration-induced RWL methods such as restricting fluids, increased exercises, sauna and training with plastic suits before competitions [8,9,10].

Although there are numerous studies related to the effect of RWL, a large number of studies did not include a control group/exercise protocol specific to judo match demands and format and were executed under the previous IJF Refereeing Rules, where athletes were weighed almost 2 h before the competition [15,24]. Under current IJF rules, the recovery period is almost 15 h following the official weigh-in [1]. To the best of our knowledge, no current study provides information on whether this time is sufficient for athletes’ recovery after RWL. Moreover, RWG has been associated with success in judo competitions [5], and its effect on sport-specific performance has not been examined in previous studies. In addition, previous research did not investigate the effect of RWL on the hydration status of judo athletes [15,24]. Furthermore, it is of great importance to understand the effects of RWL and RWG on judo-specific performance as athletes generally perform it within 2–4 days before competitions [8,25]. Therefore, this study aimed to investigate the effects of RWL and RWG on hydration status, judo-specific performance, recovery and physiological responses in highly trained judo athletes. We hypothesized that athletes would present a high level of dehydration, worse performance during judo-specific tests and worse recovery following the RWL process compared to baseline and after 15 h of recovery.

## 2. Materials and Methods

### 2.1. Participants 

Eighteen highly trained male judo athletes competing at the national and international level voluntarily participated in this study. The sample size was justified using G*Power software (Version 3.1.9.7; Universitat Kiel, Dusseldorf, Germany) with a priori test with an effect size of 0.25 and 0.85 statistical power using repeated-measures ANOVA, within–between factors test [26]. The study’s inclusion criteria were as follows: (1) at least 5 years of judo competition experience; (2) belt level of at least 1st DAN; (3) participation in international competitions such as World/European Championships, Grand Slam/Prix or continental cups; (4) free from musculoskeletal injuries in the last year. If these criteria were not met, athletes were excluded from the research. All athletes were in the preparation period during measurements. After the initial inclusion, the sample was further divided into an RWL group and a control group. The RWL group (9 athletes) included athletes that had weight-cutting experience, i.e., weight-cyclers, and had been engaged in RWL at least 5 times a year for the last 3 years. The control group (9 athletes) included athletes who did not practice weight loss before competitions and had not been involved in RWL in the last 3 years before the study. Table 1 presents the characteristics of the athletes. The study was approved by Gazi University Ethical Committee (number: 24074710-604.01.01-13). The athletes were provided with a detailed explanation of the study and gave their signed informed consent. The study was carried out according to the latest version of the Declaration of Helsinki.

### 2.2. Study Design

The current study aimed to simulate a real competition environment to test our hypothesis. Therefore, the athletes from both RWL and control groups participated in 3 measurements: anthropometry, hydration status and performance tests. The first measurement was made 48 h before reference weigh-in time (at 19:00), the same time the official weigh-in is held during official competitions. The second measurement was carried out after 48 h at 19:00 (the same as official weight-in time), during which RWL group athletes had to meet the study’s criteria of losing 5% of their body mass. Finally, the next day, the third measurement was made after 15 h of recovery (RWG) at 10:00–11:00, where the athletes of the RWL group were instructed not to gain more than 5% of their body mass. This simulated the competition environment and the official weigh-in procedures under the current IJF Refereeing Rules [1]. All athletes were instructed to refrain from caffeine and alcohol 24 h before the measurements. In addition, they were instructed to come to the baseline evaluation in a well-fed and hydrated state. Following the first evaluation, control athletes kept their routine, i.e., food and fluid intake, in the 48 h before the second measurement. RWL athletes were supposed to lose 5% of their body mass during 48 h using their usual methods to cut weight. However, they were also warned against the usage of laxatives, diuretics and diet pills. After the second measurement, athletes were instructed to maintain the same food and fluid intake as they do before competitions. RWL group was also instructed not to gain more than 5% of their body mass during the recovery following second body mass measurement according to the IJF weigh-in rule [1], which disqualifies athletes from the competition. For assessing judo performance, a special judo fitness test (SJFT) was applied in baseline measurements, at weight in (after 48 h) and after the 15 h recovery period. The SJFT was applied three times in each performance evaluation with 10 min breaks between them. The 10 min breaks also simulated the minimum official competition break that an athlete is entitled to before the next fight under the current IJF rules [1]. 

**Figure 1:**
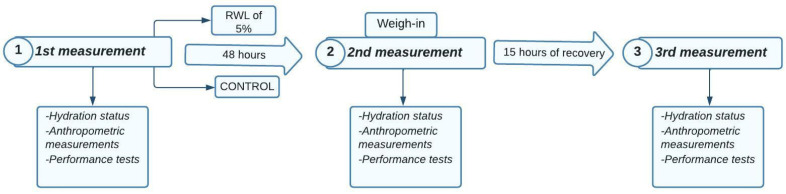
Design of the entire experimental protocol.

The summary of the study design can be found in Figure 1.

**Figure 2:**
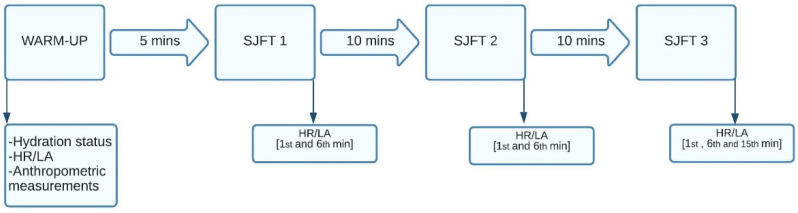
The performance test sequence. SJFT = special judo fitness test, HR = heart rate, LA = blood lactate.

The sequence of the tests performed by the athletes can be found in Figure 2.

### 2.3. Hydration Status

Urine specific gravity (USG) is an easy and reliable method to monitor the hydration status of combat sports athletes with urine concentration [27,28]. Each athlete’s urine sample was taken immediately before each body mass measurement. The samples were placed in plastic cups, and USG was determined with a digital refractometer (ATAGO PAL-10S, Tokyo, Japan) in the judo hall by the same researcher each time. As soon as the urine samples were analyzed for USG, they were immediately disposed of. The hydration status of the athletes was classified according to the American College of Sports Medicine position stand (dehydrated if USG > 1.020 g·mL^−1^) [29].

### 2.4. Body Composition

Body compositions of the athletes were determined using a bioelectrical impedance (BIA) device (Tanita, BC-545, Tokyo, Japan), which uses a dual-frequency method (50 kHz/6.25 kHz). Body composition measurements were performed in standing position, barefoot, with legs and thighs not touching. The skin and the electrodes were precleared and dried before the measurement. The general measurement guidelines [30] for BIA were followed: (1) the respondents were asked to abstain from large meals after 9 PM the evening before the test, and on the day of the measurement, they neither ate nor drank before the end of the procedure; (2) the respondents did not consume alcohol 48 h before the measurement; (3) the respondents were in the standing position for at least 5 min before the measurement to redistribute the tissue fluids; (4) hands were not touching the torso and were placed 15 cm laterally from the body. The high test–retest reliability and accuracy of BC-545 were previously assessed, with interclass correlation (ICC) reported at 0.99, and correlations with the reference measure (dual-energy X-ray absorptiometry (DXA)) were shown to be significant (r  >  0.9) [30]. Body mass, body fat percentage, muscle mass and body fat percentage were recorded for each athlete. 

### 2.5. Physiological Responses 

Resting blood lactate (LA) and heart rate (HR) of the athletes were measured following a 30 min rest in the supine position. HR and LA were also monitored at the 1st and 6th minutes following each SJFT application and 1st, 6th and 15th minutes following the last SJFT application [31]. LA was measured with a lactate device (Edge Blood Lactate Monitoring System, ApexBio Inc., Taipei City, Taiwan) by the same experienced researcher. Before each measurement, the site was cleaned with alcohol and dried with cotton, and a 0.3 µL blood sample was obtained from the fingertip of the middle finger. HR was monitored with an HR monitor (SEEGO, Realtrack Systems, Madrid, Spain). 

### 2.6. Judo-Specific Performance

SJFT was used to simulate judo match performance as it presents the same physiological responses following a judo match and also has positive relationships with performance parameters in competition (e.g., effective combat time and number of attacks) [32,33]. Three athletes of similar body weight and height performed the SJFT according to the following protocol: two judokas (uke) were positioned at a distance of 6 m from each other, while the test executor (tori) was positioned 3 m from the judokas to be thrown. The procedure was divided into three periods—15 s (A), 30 s (B) and 30 s (C)—with 10 s intervals between them. In each period, the executor threw the opponents using the *ippon-seoi-nage* technique as many times as possible. Performance was determined based on the total throws completed during each of the three periods (A + B + C). The heart rate (HR) was measured immediately after the test and then 1 min later to calculate the index using the following equation:

$$\mathrm{Index}\;(\mathrm{bpm}\cdot throws^{-1})=\frac{\mathrm{final}\;\mathrm{HR}\;(\mathrm{bpm})+\mathrm{HR}\;\mathrm{at}\;1\;\min\;\mathrm{after}\;\mathrm{the}\;\mathrm{test}\;\left(\mathrm{bpm}\right)}{\mathrm{Number}\;\mathrm{of}\;\mathrm{throws}}$$

Reliability values (ICC) of the number of throws and index have been previously reported as 0.73 and 0.88, respectively [34].

### 2.7. Statistical Analysis

The data were presented as mean ± standard deviation with 95% confidence intervals (CI). The data normality was checked with the Shapiro–Wilk test and descriptive methods using skewness and kurtosis coefficients [35]. Independent sample T-test was used to compare physical characteristics and SJFT performances between RWL and control groups. When a significant difference was found, the effect size was measured according to Cohen [36]. The effect of RWL on variables was tested with split-plot ANOVA. The effect of the experiment on USG and body mass was verified with group × test (2 × 3) factors, while its effects on SJFT performance, HR and LA were verified with group × measurement day × test (2 × 3 × 3). Sphericity was tested with Mauchly’s sphericity test, and when sphericity was violated, Greenhouse–Geisser correction was used. When a significant effect was found in repeated measures, a one-way repeated-measures ANOVA was used to determine which group or condition caused this difference. Independent sample T-test was used when there was a significant effect of group or interactions. Partial eta squared (η^2^) was used to determine effect size (ES), with 0.0099, 0.0588 and 0.1379 considered as small, medium and large effect sizes [36]. Analysis was carried out using Statistical Package for Social Science (SPSS Inc. Chicago, IL, USA) 16.0 software. Significance was set at *p* < 0.05.

## 3. Results

Table 1 presents the baseline characteristics of the participants.

Related to USG, a significant main effect of time was found (F1.7-26.8 = 6.83, η^2^ = 1.67, ES = large, *p* < 0.001) while there was no significant effect of group factor (F1-16 = 3.45, η^2^ = 0.17, *p* = 0.08, ES = large). When the difference among the measurement days was investigated, there was no significant difference in the control group (*p* > 0.05), whereas there was a significant difference in the RWL group with lower values at baseline and following recovery compared to RWL (*p* < 0.001). Although there was no significant main effect of group on USG changes, there was a significant interaction effect of time and group on USG (F1.7-26.8 = 13.2, η^2^ = 1.67, *p* < 0.001, ES = large). In other words, the differences in USG values of RWL group were found different from those obtained from the control group. Table 2 presents USG values of the athletes from RWL and control groups. 

Table 2 reports that the control group remained hydrated (USG ≤ 1.020 g·mL^−1^) throughout the measurements, and the RWL group presented dehydration (USG > 1.020 g·mL^−1^) after the RWL process and 15 h of recovery, although they started the study in a hydrated state. 

As illustrated in Table 3, there was no significant difference in body mass measured before and after RWL and following 15 h of recovery (F1.2-19.6 = 0.93, η^2^ = 0.056, *p* = 0.37, ES = small). There was also no significant main effect of group on body mass changes (F1-16 = 4.11, η^2^ = 0.20, *p* = 0.06, ES = large).

The data related to throw numbers in SJFT applied by athletes at baseline, after RWL and after 15 h of recovery are presented in Table 4. The changes in total throw numbers during SJFT applications were found similar among three measurements (F3.1-49.5 = 2.12, η^2^ = 0.12, *p* = 0.11, ES = medium). Moreover, RWL and measurement times did not affect total throw numbers during each SJFT (F1.8-29.2 = 1.66, η^2^ = 0.09, *p* = 0.21, ES = medium; F1-16 = 1.15, η^2^ = 0.07, *p* = 0.30, ES = medium, respectively). However, a significant main effect of time on SJFT total throw numbers was found (F1.9-30.4 = 7.70, η^2^ = 0.33, *p* < 0.001, ES = large). 

According to one-way repeated-measures ANOVA results, the RWL group showed worse performance following RWL with lower throw numbers after RWL compared to after 15 h of recovery (*p* < 0.05). 

SJFT index did not differ between groups among different measurement times at baseline, after RWL and after 15 h of recovery (F2.9-46.3 = 1.24, η^2^ = 0.07, *p* = 0.31, ES = medium). SJFT index differed between groups and at different measurement times (F1.7-26.4 = 8.05, η^2^ = 0.33, *p* = 0.01, ES = large; F1-16 = 6.43, η^2^ = 0.29, *p* = 0.02, ES = large, respectively), while it did not differ among three SJFTs implemented consecutively (F1.3-21.4 = 2.01, η^2^ = 0.11, *p* = 0.17, ES = medium). According to one-way repeated-measures ANOVA results, there was significant effect of RWL group on changes in SJFT index (F2.3-24.6 = 4.87, η^2^ = 0.38, *p* = 0.02, ES = large). The differences related to SJFT index between measurements after RWL and after recovery in RWL group can be seen in Table 5.

LA changes were not affected by time, test and group factors (F5.6-89.8 = 1.66, η^2^ = 0.09, *p* = 0.15, ES = medium). Moreover, there was no significant interaction effect of time and test, time and group, and test and group factors (F5.61-89.8 = 1.43, η^2^ = 0.08, *p* = 0.21, ES = medium; F1.54-24.63 = 0.64, η^2^ = 0.04, *p* = 0.50, ES = small; F4.22-67.70 = 0.90, η^2^ = 0.05, *p* = 0.47, ES = small, respectively). While LA did not differ between groups at baseline, after RWL and after 15 h of recovery (F1.54-24.63 = 1.55, η^2^ = 0.09, *p* = 0.23, ES = medium; F1-16 = 0.04, η^2^ = 0.01, *p* = 0.90, ES = medium, respectively), it differed during SJFT applications implemented consecutively (F4.22-67.65 = 130.48, η^2^ = 0.89, *p* = 0.00).

HR did not differ between groups following each SJFT at baseline, after RWL and after 15 h of recovery (F5.15-82.43 = 0.97, η^2^ = 0.06, *p* = 0.44, ES = medium). Moreover, there was no significant interaction effect of time and group or test and group factors (F1.98-31.67 = 1.42, η^2^ = 0.82, *p* = 0.26, ES = large; F2.82-45.15 = 1.90, η^2^ = 0.10, *p* = 0.15, ES = medium). However, HR differed following each SJFT performance at baseline, after RWL and after 15 h of recovery (F5.15-82.43 = 143.10, η^2^ = 0.90, *p* = 0.00, ES = large). In addition, HR showed difference at baseline, after RWL and after 15 h of recovery (F1.98-31.67 = 31.14, η^2^ = 0.66, *p* = 0.00, ES = large). Furthermore, there was significant main effect of test and group factors separately on HR changes (F2.82-45.15 = 313.00, η^2^ = 0.95, *p* < 0.001, ES = large; F1-16 = 10.50, η^2^ = 0.40, *p* = 0.01, ES = large, respectively). 

**Figure 3:**
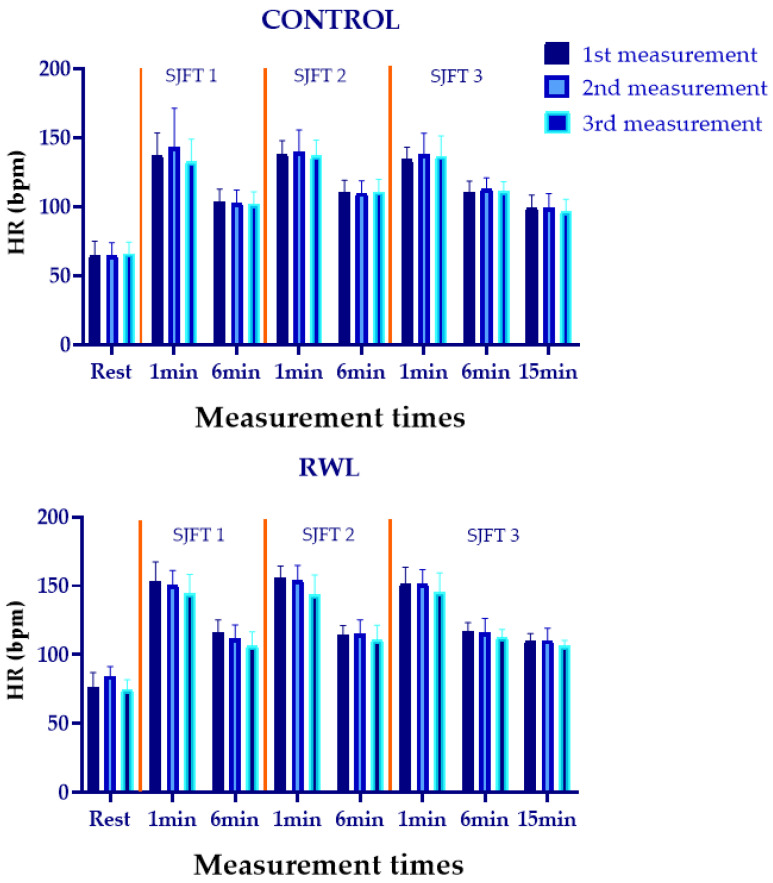
HR changes in RWL and control groups during SJFT performance and recovery.

Following RWL, a significant difference was found in HR values between RWL and control groups. RWL group presented higher HR values 1 min after the second SJFT compared to the control group (control = 143 bpm vs. RWL = 150 bpm, *p* = 0.04). RWL group also presented higher values 1 min after the third SJFT performance (control = 137 bpm vs. RWL = 151 bpm, *p* = 0.03). HR changes can be found in Figure 3.

## 4. Discussion

This study aimed to investigate the effect of RWL and recovery on hydration status, judo-specific performance and physiological responses such as HR and LA in elite judo athletes. The main findings of the study were that (1) RWL resulted in a high level of dehydration in the RWL group, which did not change even after 15 h of recovery and 5% weight gain, while the hydration level of the control group stayed unchanged throughout the measurements; (2) the RWL group presented better performance following 15 h of recovery compared to second measurements following RWL; and (3) the RWL group demonstrated higher HR values during recovery following RWL compared to control group.

Fluid loss and decreasing fluid intake are the most applied weight loss methods among combat sports athletes [10,25]. In the current study, the RWL group presented a higher level of dehydration following RWL than the control group. Moreover, they did not rehydrate enough to be classified as hydrated before the third measurement and presented dehydration again. Previous research related to the hydration status of judo athletes demonstrates that dehydration is common in judo athletes from different age categories during training and competition [20,21,37]. Ceylan et al. [37] investigated the hydration status of high-level judo athletes before and after a competition and stated that athletes remained dehydrated starting from a week before the competition and 24 h after the competition. In another study investigating the effect of weigh-in time on the hydration status of combat sports athletes, Ceylan et al. [38] highlighted that judo athletes could not recover from weigh-in to pre-competition and presented a high level of dehydration in the competition morning compared to wrestlers who had only 2 h for recovery. The case is the same during off-season training, as presented by Stefanovsky et al. [23]. The authors stated that athletes presented non-optimal hydration status when they trained in an off-season camp and did not have to lose weight. Thus, it can be concluded that dehydration is a common practice in judo athletes, which should be addressed promptly to preserve judo athletes’ health. In addition, the elite athletes involved in RWL are typically weight cycling many times in a year, with their weight fluctuating dramatically between the competitive and off seasons [39,40], and particularly between competitions. Research has suggested that athletes engaging in chronic weight cycling may adapt to the stresses associated with RWL [41]. That would mean that athletes could become more resistant to its adverse effects on performance. This could also affect the sensitivity of the scientific tests they undergo to detect actual effects of RWL. However, this still needs to be further researched in elite judo. 

Previous research demonstrated that insufficient recovery following dehydration led to decrements in aerobic and anaerobic performance and thus led to worse performance [42,43,44]. When hydration status of RWL athletes following 15 h of recovery is considered, athletes could not compensate for fluid loss during recovery and could not present a USG value lower than 1.020. However, RWL-induced dehydration did not negatively affect the consecutive SJFT performance of RWL athletes in the current study. In contrast to our findings, previous research related to the effect of dehydration on performance in combat sports suggested that acute dehydration induced decreases in muscle strength–endurance, glycolytic energy pathway and physical performance tests and an increase in fatigue [13,45,46].

SJFT has the same physiological responses following a judo match and also has positive relationships with performance parameters in competition (e.g., effective combat time and the number of attacks) [32,33] and comprises high-intensity efforts, and thus the contribution of anaerobic metabolism is high. Although changes in SJFT performance between control and RWL groups were found similar in the current study, when the RWL group was investigated separately, their SJFT index following 15 h of recovery was found better compared to that calculated after RWL. There are conflicting results related to the effect of RWL and dehydration in the literature. Some studies noted that dehydration led to decreased anaerobic peak power and lower and upper extremity Wingate performance and increased fatigue index [47,48], while others indicated no decrements in performance. In contrast to our findings, Fortes et al. [49] did not observe any change in the SJFT index after RWL in judo athletes. However, they indicated that athletes performed decreased SJFT total throws following RWL. Although there was no decrease in the SJFT index of RWL athletes, an increase in physiological responses during the exercise was monitored, which was also observed in the current study. It can be explained by increased blood viscosity which decreases cardiac output and HR recovery following a particular workload [50]. 

In practice, RWL should be avoided as much as possible to avoid increased strain on the cardiovascular system. Therefore, coaches and athletes (C&A) should pay more attention to better pre- and in-season weight management. In addition, C&A should strive to reduce weight cycling and the difference between training and competition bodyweight as much as possible. C&A should also focus their attention on developing personalized strategies of RWG to achieve proper regeneration and hydration status before the first fight. They should keep in mind that RWG of 5% in 15 h before the competition does not equal a normal body hydration status. Future studies should also try to address the aforementioned issues. 

This study had some limitations. The food and fluid intakes of the athletes were not recorded and monitored throughout the study. It would provide an insight into the recovery process of judo athletes between weigh-in and the start of the competition if the food and fluid intakes of the athletes were recorded. In addition, athletes trained during the 48 h of the weight-cutting process, but training intensities and weight-cutting methods of the athletes were not monitored and were self-reported. Future studies could include such variables. Additionally, the fact that BIA provides accurate results depends on the guidelines proposed by the manufacturer; however, they could not be completely followed due to the nature of the study with a simulation of the competition environment. 

## 5. Conclusions

RWL procedures induced dehydration in judo elite athletes, and the athletes could not rehydrate between RWL and the last measurement following 15 h of recovery. This study showed that RWL did not affect judo-specific performance but partially increased physiological load during the exercise. However, it is important to note that athletes did not rehydrate enough even after 15 h of recovery and despite up to 5% weight gain. Therefore, future studies are warranted to focus on the effects of RWL and long-term dehydration by monitoring athletes’ performance changes as well as health-related variables.

## Figures and Tables

**Table 1:** Baseline characteristics and judo-specific performances of the athletes.

Variables	Control (n = 9)	%95 CI	RWL (n = 9)	%95 CI	*p*
Age (years)	19.1 ± 1.2	(18–20)	19.8 ± 2.7	(18–21)	0.45
Body height (cm)	176.7 ± 3.6	(176–179)	172.1 ± 5.9	(167–179)	0.06
Body mass (kg)	77.5 ± 10.8	(69.2–85.8)	71.2 ± 9.3	(64.0–78.4)	0.20
Fat-free mass (kg)	65.6 ± 6.4	(60.7–70.5)	61.5 ± 7.3	(55.9–67.1)	0.22
BMI (kg/m^2^)	24.78 ± 3.17	(22.3–27.2)	23.99 ± 2.31	(22.2–25.7)	0.55
BFP (%)	10.6 ± 4.7	(7.02–14.31)	8.4 ± 2.9	(6.2–10.6)	0.25
SJFT A (throw numbers)	6 ± 0.0	(5.6–6.3)	6 ± 0.5	(5.6–6.3)	1.00
SJFT B (throw numbers)	11 ± 0.7	(10.2–11.4)	11 ± 0.3	(10.8–11.3)	0.45
SJFT C (throw numbers)	10 ± 0.7	(9.5–10.7)	10 ± 1.0	(8.9–10.4)	0.31
SJFT (total throw numbers)	27 ± 1.0	(25.9–28.0)	27 ± 1.0	(25.7–27.7)	0.73
SJFT A HR (bpm)	155 ± 6	(144–168)	164 ± 3	(157–171)	0.17
SJFT B HR (bpm)	169 ± 4	(161–178)	185 ± 2	(180–191)	0.01
SJFT C HR (bpm)	172 ± 1	(163–181)	187 ± 2	(181–192)	0.01
SJFT 1 min HR (bpm)	137 ± 5	(123–150)	153 ± 5	(143–164)	0.04
SJFT index (score)	11.4 ± 0.7	(10.9–12.0)	12.7 ± 0.9	(12.0–13.4)	0.01

BMI = body mass index; BFP = body fat percentage; SJFT = special judo fitness test; A, B, C = parts of the SJFT; HR = heart rate.

**Table 2:** Changes in USG values in RWL and control groups among measurements.

Measurement Day	RWL	%95 CI	Control	%95 CI	Factors		
					Time	Group	Time × Group
Measurement 1	1.014 ± 0.005	(1.011–1.019)	1.019 ± 0.006	(1.015–1.024)			
Measurement 2	1.026 ± 0.003	(1.024–1.030)	1.017 ± 0.004	(1.014–1.021)	6.82 *	3.45	13.2 *
Measurement 3	1.022 ± 0.005	(1.018–1.026)	1.017 ± 0.003	(1.015–1.020)			

RWL = rapid weight loss group, CI = confidence interval. * *p* < 0.05.

**Table 3:** Changes in body mass in RWL and control groups among measurements.

Measurement Day	RWL	%95 CI	Control	%95 CI	Factors		
					Time	Group	Time × Group
Measurement 1	71.2 ± 9.3	(64.0–78.4)	77.5 ± 10.8	(69.2–85.8)			
Measurement 2	67.7 ± 9.0	(60.0–74.7)	77.6 ± 11.0	(69.1–86.1)	0.93	4.10	1.06
Measurement 3	67.8 ± 5.5	(63.5–72.0)	77.6 ± 11.2	(68.9–86.2)			

RWL = rapid weight loss group, CI = confidence interval.

**Table 4:** Changes in SJFT throw number between control and RWL groups.

		Control		RWL			F	
	Tests	Mean ± SD	%95 CI	Mean ± SD	%95 CI	Time	Group	Time × Group
	SJFT a	27 ± 1	(25–30)	27 ± 1	(25–29)			
Measurement 1	SJFT b	28 ± 1	(26–30)	27 ± 1	(25–27)			
	SJFT c	28 ± 2	(24–30)	27 ± 2	(25–29)			
	SJFT a	28 ± 1	(26–30)	27 ± 2	(23–29)			
Measurement 2	SJFT b	27 ± 2	(26–32)	26 ± 2	(23–28)	7.70 *	1.66	2.12
	SJFT c	28 ± 2	(26–31)	26 ± 2	(24–29)			
	SJFT a	28 ± 2	(26–32)	27 ± 2	(25–31)			
Measurement 3	SJFT b	28 ± 2	(26–32)	28 ± 2	(26–31)			
	SJFT c	28 ± 2	(27–32)	29 ± 2	(26–33)			

SJFT = special judo fitness test; a, b, c = parts of the SJFT, * *p* < 0.05.

**Table 5:** Changes in SJFT index between measurements after RWL and after 15 h recovery of RWL group.

SJFT Indexes				
After RWL of 5%		After Recovery		*p*
SJFT 1		SJFT 1	11.86 ± 0.89	0.05
	12.47 ± 1.13	SJFT 2	11.61 ± 1.01	0.04
		SJFT 3	11.45 ± 1.00	0.03
SJFT 2		SJFT 1	11.86 ± 0.89	0.01
	12.84 ± 1.18	SJFT 2	11.61 ± 1.01	0.01
		SJFT 3	11.45 ± 1.00	0.00
SJFT 3		SJFT 1	11.86 ± 0.89	0.01
	12.90 ± 1.07	SJFT 2	11.61 ± 1.01	0.01
		SJFT 3	11.45 ± 1.00	0.00

SJFT = special judo fitness test.

## Data Availability

The datasets used and/or analyzed during the current study are available from the corresponding author on reasonable request.
